# Tissue dose estimation after extravasation of ^177^Lu-DOTATATE

**DOI:** 10.1186/s40658-021-00378-3

**Published:** 2021-03-31

**Authors:** Perrine Tylski, Géraldine Pina-Jomir, Claire Bournaud-Salinas, Patrice Jalade

**Affiliations:** 1 Service de Physique Médicale et Radioprotection, Hospices Civils de Lyon, Lyon, France; 2 Service de Médecine Nucléaire, Groupement Hospitalier Est, Hospices Civils de Lyon, Lyon, France

**Keywords:** Extravasation, Dosimetry, ^177^Lu-DOTATATE

## Abstract

**Background:**

Extravasation of radiopharmaceuticals used for vectorized internal radiotherapy can lead to severe tissue damage (van der Pol et al., Eur J Nucl Med Mol Imaging 44:1234–1243, 2017). Clinical management of these extravasations requires the preliminary estimation of the dose distribution in the extravasation area. Data are scarce regarding the dose estimation in the literature. This work presents a methodology for estimating the dose distribution after an extravasation occurred in September 2017, in the arm of a patient during a 7.4-GBq infusion of Lutathera ® (AAA).

**Methods:**

A local quantification procedure initially developed for renal dosimetry was used. A calibration factor was determined and verified by phantom study. Extravasation volume of interest and its variation in time were determined using 4 whole body (WB) planar acquisitions performed at 2 h (*T*_2h_), 5 h (*T*_5h_), 20 h (*T*_20h_), and 26 h (*T*_26h_) after the beginning of the infusion and three SPECT/CT thoracic acquisitions at *T*_5h_, *T*_20h_, and *T*_26h_. For better estimation of initial extravasation volume, 3 volumes were defined on SPECT images using a 3D activity threshold. Cumulated activities and associated absorbed doses (*D*_1_, *D*_2_, *D*_3_) were calculated in the 3 volumes using the MIRD formalism.

**Results:**

Volumes estimated using 3D threshold were *V*_1_ = 1000 mL, *V*_2_ =400 mL, and *V*_3_ =180 mL. Cumulated activities were evaluated using a monoexponential fit on activities calculated on SPECT images. Estimated local absorbed doses in *V*_1_, *V*_2_, and *V*_3_ were *D*_1_ = 2.3 Gy, *D*_2_ = 4.1 Gy, and *D*_3_ = 6.8 Gy. Evolution in time of local activity in the extravasation area was consistent with an effective local half-life (*T*_eff_) of 2.3 h.

**Conclusions:**

Rapid local dose estimation was permitted thanks to knowledge of the calibration factor determined previous to accidental extravasation. Lutathera® lymphatic drainage was quick in the arm (*T*_eff_ = 2.3h). Estimated doses were in the lower range of deterministic effects and far under soft tissue necrosis threshold. Thus, no surgical rinse was proposed. The patient did not show any clinical consequence of the extravasation.

## Background

[^177^Lu-DOTA,Tyr(3)]octreotate (^177^Lu-DOTATATE) is an effective treatment of advanced well differentiated gastro-entero-pancreatic neuro-endocrine tumors (GEP NET) [1]. It relies on the combination of ^177^Lu, a medium energy beta-emitter, with a peptide, binding specifically to tumors. ^177^Lu emits beta radiation with a mean energy of 133 keV (79%), photonic radiations (γ) of 113 keV (6%) and 208 keV (10%), and a half-life of 6.65 days [2]. Its physical characteristics are appropriate for tumor treatment, gamma-camera imaging, and dosimetry calculation.

^177^Lu-DOTATATE is used in clinical routine in several countries for GEP NET. It received US FDA approval in January 2018 and EMA approval in September 2017. It is administered intravenously during 10–30 min [3]. As for any intravenous administration, extravasation may occur during ^177^Lu-DOTATATE injection. The retention of beta-emitting radiopharmaceutical in soft tissues can lead to very severe tissue damage [4].

Dosimetric evaluations are rare in extravasation cases [4] and extravasation cases of ^177^Lu radiopharmaceutical are sparse in the literature. Two extravasation cases of ^177^Lu-PSMA-617 have been documented. One case was presented as a commented image of the patient 2, 20, and 40 h post injection [5] and the second is a brief case report with low details on dose calculation [6]. One case of ^177^Lu –DOTATOC extravasation in the arm has been recently published, using SPECT images and a survey meter to determine an effective half-life [7].

In September 2017, during an intravenous administration of a first cycle of Lutathera® (Advanced Accelerator Applications) treatment in our center, extravasation occurred in the arm of the patient. The radioactive solution accumulated in the upper part of the patient’s arm. SPECT/CT and whole body images were acquired and absorbed doses were estimated.

## Methods

### Initial phantom study

Activity estimation in the extravasation area of the patient images was realized using a local quantification protocol initially developed for renal dosimetry with ^177^Lu. Two phantom studies had been performed prior to the incident: one to set a calibration factor F in counts/MBq/s for ^177^Lu and one to evaluate its accuracy on SPECT/CT images.

**Fig. 1:**
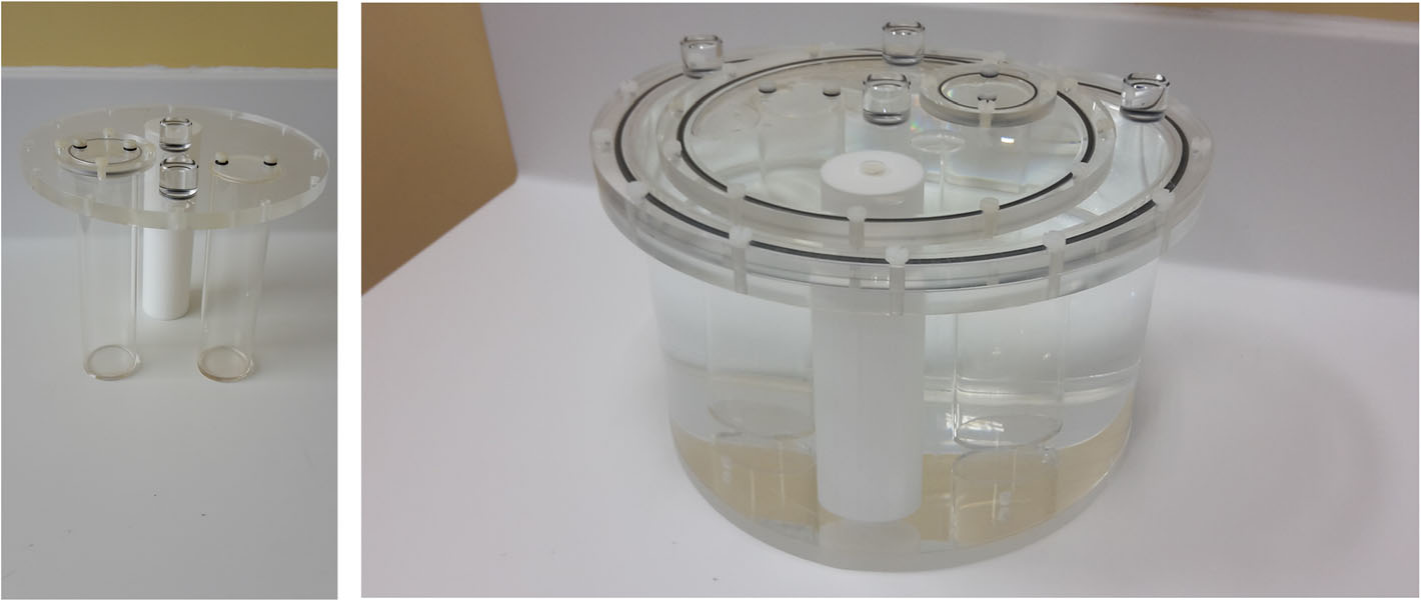
3-cylinder cover (left), and calibration phantom with body phantom and 3-cylinder cover (right)

The calibration phantom is a body shape phantom (PTW Body phantom “B”) with a cover containing 3 cylindrical inserts (PTW cover D with 3 cylinders), of which 2 are fillable and one is made of polytetrafluoroethylene to simulate bone. The inner diameter of the cylinders was 4.6 cm. Volumes of the fillable cylinders were determined by weighing and were respectively 303 (± 0.5 mL) and 321 (± 0.5 mL) mL. The volume of the inserts was slightly larger, yet in the range of magnitude of normal kidney volume, 202 mL for men and 154 mL for women [8]. The two cylinder inserts (Fig. 1) were filled with ^177^Lu-DOTATATE and placed in the body shape phantom filled with water without activity.

**Fig. 2:**
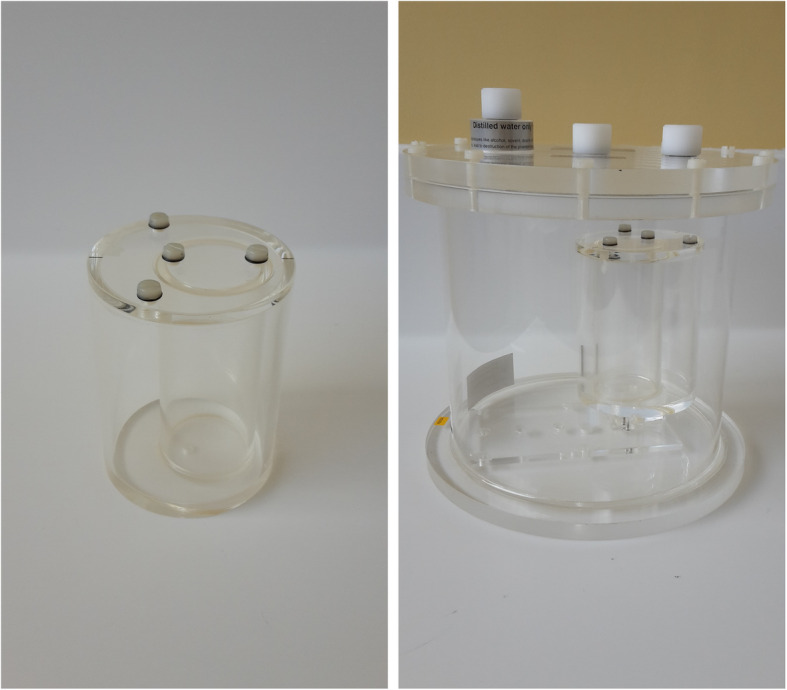
Heart phantom insert (left), and an example of a verification phantom setup with the heart insert placed in a head phantom (right)

Two verification phantoms were used (Fig. 2): a cylindrical phantom (PTW Head Phantom “H”) and the body shape phantom previously described. The phantoms were alternatively changed from one acquisition to the other to introduce more variability in the verification phase. A heart insert made of two non-axial cylinders was used (PTW Heart phantom “C”). Only the inner cylinder, with an inner diameter of 4.4 cm and a length of 10 cm (weighted volume 150 ± 0.5 mL) was filled with ^177^Lu-DOTATATE. The outer cylinder was filled with water and placed in the cylinder or body phantom filled with water.

A reference activity concentration (AC) of 1 MBq/mL was considered, corresponding to a value measured in a patient kidney 24 h after ^177^Lu-DOTATATE, as reported in the literature [9]. Six acquisitions around 7 days apart were performed for each phantom, to cover a wide range of activity concentration (AC) around this reference value. All images were acquired on a Symbia T2® camera (Siemens Healthcare) with a 5/8″ crystal and a MELP collimator. Images were acquired and reconstructed according to the parameters detailed in Tables 2 and 3, which were determined using joint EANM/MIRD recommendations for quantitative ^177^Lu SPECT [2]. Images were corrected for attenuation using CT and for scatter using a double energy window method.

Activities were determined using a radionuclide calibrator, calibrated for ^177^Lu (MEDI 405®, Veenstra) with an uncertainty of 2.6% by a laboratory (CERCA LEA) traceable to the national primary laboratory (Henry Becquerel National Laboratory). The total activities in the calibration and verification inserts and the corresponding activity concentrations are reported in Table 1.

**Table 1:** Activities and activity concentrations in the cylinders used for calibration and verification

Acquisition	Calibration-cylinder 1		Calibration-cylinder 2		Verification-cylinder	
	A (MBq)	AC (MBq/mL)	A (MBq)	AC (MBq/mL)	A (MBq)	AC (MBq/mL)
1	2275 ± 59	7.51 ± 0.20	1136 ± 30	3.54 ± 0.09	471 ± 12	3.14 ± 0.08
2	1700 ± 44	5.61 ± 0.15	849 ± 22	2.65 ± 0.07	354 ± 9	2.36 ± 0.06
3	808 ± 21	2.67 ± 0.07	403 ± 10	1.26 ± 0.03	167 ± 4	1.12 ± 0.03
4	388 ± 10	1.28 ± 0.03	194 ± 5	0.60 ± 0.02	80 ± 2	0.54 ± 0.01
5	186 ± 5	0.61 ± 0.02	93 ± 2	0.29 ± 0.01	39 ± 1	0.26 ± 0.01
6	90 ± 2	0.30 ± 0.01	45 ± 1	0.140 ± 0.004	19 ± 0.5	0.12 ± 0.003

Images were processed using Matlab® (Mathworks). The number of counts was calculated in a volume of interest (VOI) equal to the known volume of the cylinders on the calibration images, with a precision of one voxel (0.064 mL). The number of counts was divided by the acquisition duration and plotted against the activity. The calibration factor F in counts/s/MBq was determined using a linear regression on this plot and uncertainty was determined using EANM practical guidance on uncertainty analysis for dose calculation [10].

In the verification step, we tried to simulate a patient image processing: VOI was manually determined slice by slice on the CT images of the phantom and reported on SPECT images. The number of counts in the VOI was calculated and converted into activity using F. The calculated activity was compared to the known activity.

### Patient

Extravasation occurred during the first cycle of treatment in a 70-year-old male patient, previously operated for small intestine neuro-endocrine tumors, with several metastatic lesions in the liver, bone lesions, and one subclavicular lymph node, all expressing somatostatin receptors on 111In-pentreotide scintigraphy. Nephroprotective amino acids solution was perfused intravenously in the right arm from 10:40 without any incident. Lutathera was injected in the left median cubital vein at 11:40 (*T*_0_). The routine injection protocol was used, using a pump, pushing the saline solution in the vial containing the ^177^Lu-DOTATATE, which was pushed in a second manifold to the vein of the patient, by the pressure increase in the vial. The flow was set to 100 mL/h, and then increased to 200 and 300 mL/h. At the end of the infusion at 12:40, the nurse noticed a swelling just over the left elbow. The patient did not complain of any pain. The nurse immediately stopped the infusion and informed the nuclear physician, who confirmed the extravasation. The medical physicist and the local referee for nuclear and radiological incidents were informed. The residual activity of Lutathera in the vial was measured using a dose calibrator, calibrated for ^177^Lu (MEDI 405®, Veenstra).

Osmogel® dressings were placed on the injection site. The patient underwent several whole body and SPECT/CT images. The local procedure relying on national guidelines for radiopharmaceutical extravasation was followed [11]. Adapted interventions were used to stimulate the lymphatic elimination during several hours after the detection of the extravasation, including in the night: warming and elevation of the left arm and repeated self-massaging.

### Imaging

Whole body images were acquired with a spectrometric window of 208 keV ± 10% and a 20 cm/min speed. The first whole body image was acquired 2 h (*T*_2h_) after the beginning of the infusion and 1 h after the extravasation was detected. Following routine acquisition protocol, the patient urinated before this first acquisition.

A second whole body image was acquired 5 h (*T*_5h_) after the beginning of the infusion. This WB image was followed by a SPECT/CT acquisition on the arm. All SPECT/CT images were acquired with the parameters provided in Table 2 and reconstructed using the parameters specified in Table 3. Scatter and CT-based attenuation corrections were applied.

**Table 2:** Acquisition parameters for SPECT acquisitions

	SPECT acquisition
Spectrometric window	Main, 208 keV ± 7.5%; scatter, 180 keV ± 6%
Acquisition conditions	128 × 128 matrix, 2 × 30 projections of 30 s, auto-contour

**Table 3:** Reconstruction parameters for SPECT images

Reconstruction algorithm	Flash 3D ®
Iterations	10
Subsets	10
Post-filtering	Gaussian filter with 4 mm FWHM

The first two whole body acquisitions and the SPECT/CT images were used to make a preliminary estimation of the dose to the arm, a necessary step for deciding whether surgical rinse might be performed to avoid irreversible deterministic effects to the patient, such as tissue necrosis.

**Fig. 3:**
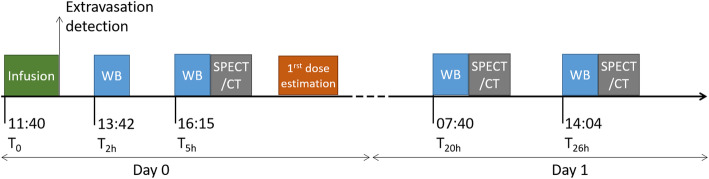
Chronology of image acquisitions from the beginning of infusion

The next morning, a WB acquisition was performed at 7:40 (*T*_20h_), followed by a SPECT/CT acquisition. A last imaging session was realized at 14:04 (*T*_26h_) with WB and SPECT/CT images to refine the first dose estimation made on the day of the incident. Imaging times and types are shown diagrammatically on Fig. 3.

### Volume estimation

After extravasation, the ^177^Lu-DOTATATE spread heterogeneously in the subcutaneous tissue. Due to very low contrast, CT could not be used to determine the extravasation volume. For the initial dose estimation, the extravasation volume was determined using a 3D threshold on a ®Syngo Via software (Siemens).

For the second dose estimation, in order to obtain information on the dose distribution, three volumes of interest were defined: one large volume encompassing the extravasation area and including low uptake voxels, one medium volume close to the volume used for the initial dose estimation, and one smaller volume corresponding to the voxels with higher uptake. The corresponding thresholds used were 4%, 10%, and 21% of the maximum intensity value in the extravasated area. These low threshold values are explained by the non-homogeneous uptake in the extravasated area. This approach was used to obtain a range of dose estimations, more informative than one single value.

### Dose calculation

The MIRD formalism was used to determine the dose in the extravasated area [12]. Given the low energy of beta emissions from ^177^Lu and the high uptake of the extravasation region, only self-irradiation was considered for dose calculation.

For the initial dose estimation using the first two time points, the percentage of activity in the arm related to the activity in the whole body was used to estimate the activity in the extravasated area, neglecting the activity eliminated in the urine of the patient. This calculation was made on the geometrical mean on anterior and posterior WB *T*_2h_ images. WB images were processed with ImageJ v1.51n [13] and SPECT/CT images were processed on Syngo® software (Siemens). *T*_5h_ SPECT/CT images were used to determine the volume of the extravasated area. Effective half-life was determined on ROI drawn around the arm on the geometrical mean of the *T*_2h_ and *T*_5h_ WB images.

For the second more accurate dose estimation taking all images into account, activity in the extravasated area was determined using the calibration factor derived from phantoms data for SPECT images. VOI were defined on SPECT images and uncertainties were derived.

As no SPECT/CT images were acquired at *T*_2h_, to estimate of the activity at this time point, a ROI around the extravasation was drawn on geometric mean of whole body images acquired at *T*_2h_ and *T*_5h_ and the ratio of counts in the ROIs at *T*_2h_ and *T*_5h_ was calculated. This process was repeated 9 times to obtain 10 estimates of this ratio. The mean ratio was multiplied by the activity estimated on the SPECT CT at *T*_5h_ to approximate the activity at *T*_2h_, as shown in Eq. (1). The uncertainty of the ratio was determined using the 10 estimates.

1 $$ {A}_{SPEC{T}_{T2h}\ast }={A}_{SPEC{T}_{T5h}}\frac{Nb\  counts\  ROI\ {arm}_{W{B}_{T2h}}\ }{\  Nb\  counts\  ROI\ {arm}_{W{B}_{T5h}}} $$

The effective half-life was determined from the activity estimated on *T*_5h_ SPECT images and A_SPECT T2h*_.

For both dose estimations, cumulated activity was determined as the area under the curve of the monoexponential fit of the calculated activity.

For dose calculation, we used the approach detailed by Sandström et al. for kidney ^177^Lu-DOTATATE dosimetry based on a dose factor (DF), which is the absorbed energy per time-integrated activity concentration in nGy kg/(MBq s) [9]. DF factors calculation are based on Radar website data [14]. DF values are between 23.9 and 24.8 nGy kg/(MBq s) for spheres between 100 and 2000 g, making it a little dependent of the emission volume. We chose to use a unique approximate value of 24.35 ± 0.45 nGy kg/(MBq s) for dose estimations whereas this uncertainty may be higher due to the non-spherical shape of the activity distribution volume.

## Results

### Phantom study

**Fig. 4:**
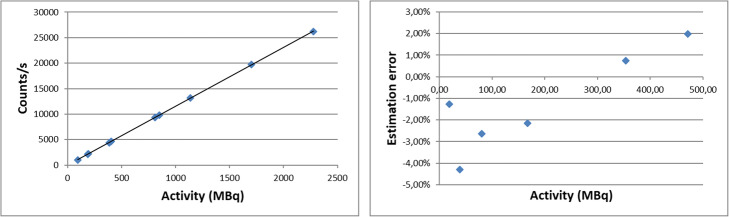
Counting rate versus activity for the calibration step (left) and error in activity estimation for the verification step (right)

The relationship between the counting rate in SPECT images and activity in the cylinder is well fit using a linear curve (Fig. 4, left). The slope of the straight line gives a calibration coefficient of 11.55 counts/s/MBq ± 0.3.

Activity in the verification phantom was estimated using this calibration factor and counts in VOI determined on CT images (Fig. 4). The average error between estimated activity in the images and activity in the cylinder measured with dose calibrator is − 1.27% (min − 4.30%, max 1.98%).

### Patient preliminary dose estimation

**Fig. 5:**
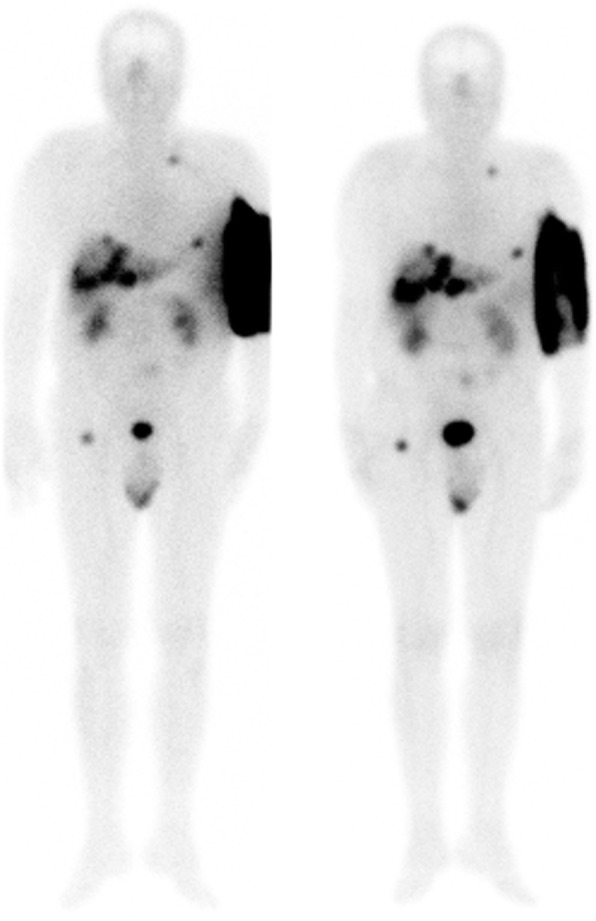
Geometric mean of whole body images acquired at *T*_2h_ (left) and *T*_5h_ (right)

**Fig. 6:**
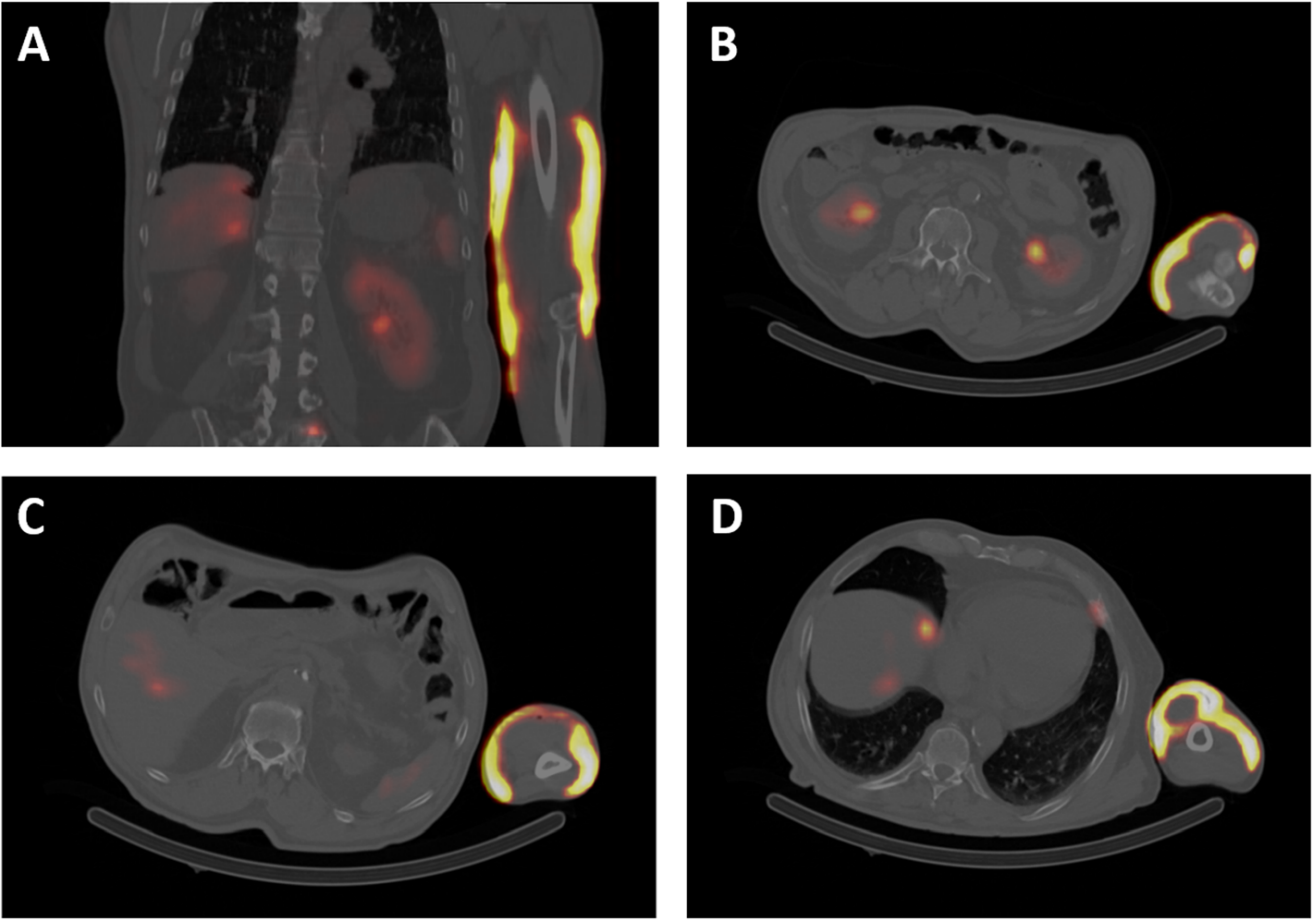
Coronal slice (**a**) and axial slices (**b**–**d**) of *T*_5h_ SPECT/CT showing the subcutaneous distribution of the extravasation in the arm

The residual activity in the vial after infusion measured with a radionuclide calibrator was 226 MBq in 20 mL. Thus, the total activity delivered to the patient was 7569 ± 203 MBq at the time of injection. A preliminary dose estimation was performed using whole body image at *T*_2h_ and *T*_5h_ (Fig. 5), and SPECT/CT images at *T*_5h_ (Fig. 6), to determine whether a surgical rinse of the arm was necessary.

Unfortunately, the extravasated area was not entirely in the field of view of the first acquisition (Fig. 5, left). The number of counts outside the field of the first image was roughly estimated using *T*_5h_ WB image. This image was truncated to get a similar truncation as *T*_2h_ WB image, and the ratio of counts in the arm area in the truncated and in the whole image was calculated. The count loss due to truncation in the first image was estimated to 5.0 ± 1.5 % and accounted for in the count calculation.

Using the first whole body image and correcting for truncation, 74% of the injected ^177^Lu activity was concentrated in the upper part of the arm compared to the whole body. A monoexponential curve was fitted based on the counts measured on the arm and whole body counts at *T*_2h_ and *T*_5h_. The effective half-life was around 1.90 h in the arm and 4.36 h in the whole body. Given the risk of necrosis for the patient and the lack of reliability of a monoexponential fit on 2 points, we chose to use the largest of these computed effective half-lives for evaluating the dose in the arm, namely the whole body half-life.

An extravasated volume of around 400 mL was estimated based on *T*_5h_ SPECT/CT image. The evaluated dose was around 8 Gy. This value was lower than the threshold for deterministic interstitial tissue necrosis in extravasation case (around 20 Gy) [15] and eliminated the need for a surgical rinse of the arm of the patient. Nevertheless, adapted interventions were applied to the patient: the nurses warmed and elevated his arm during the afternoon and the evening after the extravasation. The patient performed repeated self-massages of the upper part of the arm in the evening and in the night.

### Second estimation of activity from SPECT/CT images after extravasation

**Fig. 7:**
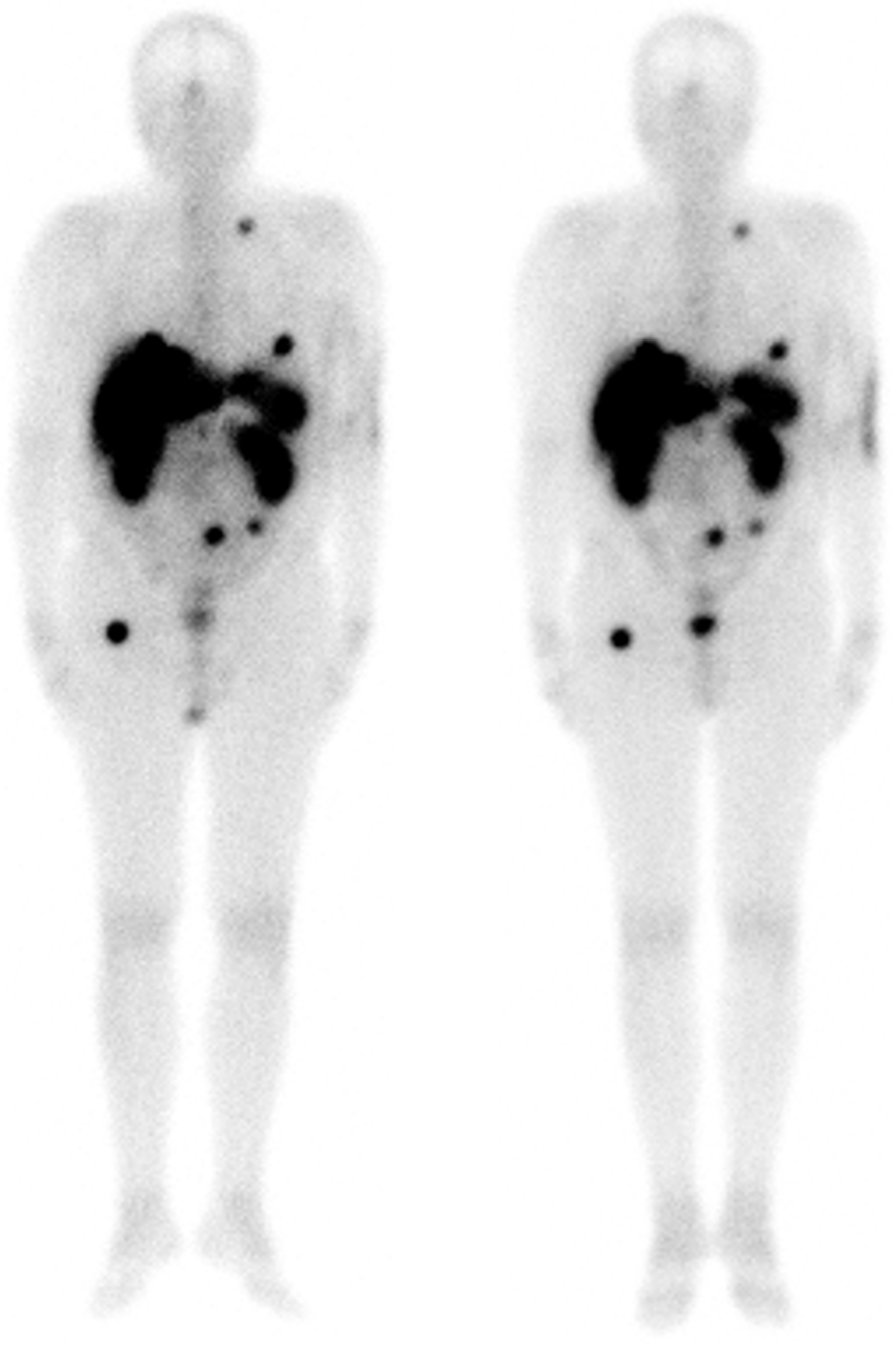
Geometric mean of whole body images acquired at *T*_20h_ (left) and *T*_26h_ (right)

Whole body (Fig. 7) and SPECT/CT images acquired the day after extravasation allowed a more precise evaluation of the dose in the arm and show a reduction of the extravasation in the arm.

The effective half-life calculated using counts on *T*_2h_, *T*_5h_, *T*_20h_, and *T*_26h_ was 3.5 h in the arm and 10.5 h in the whole body.

**Fig. 8:**
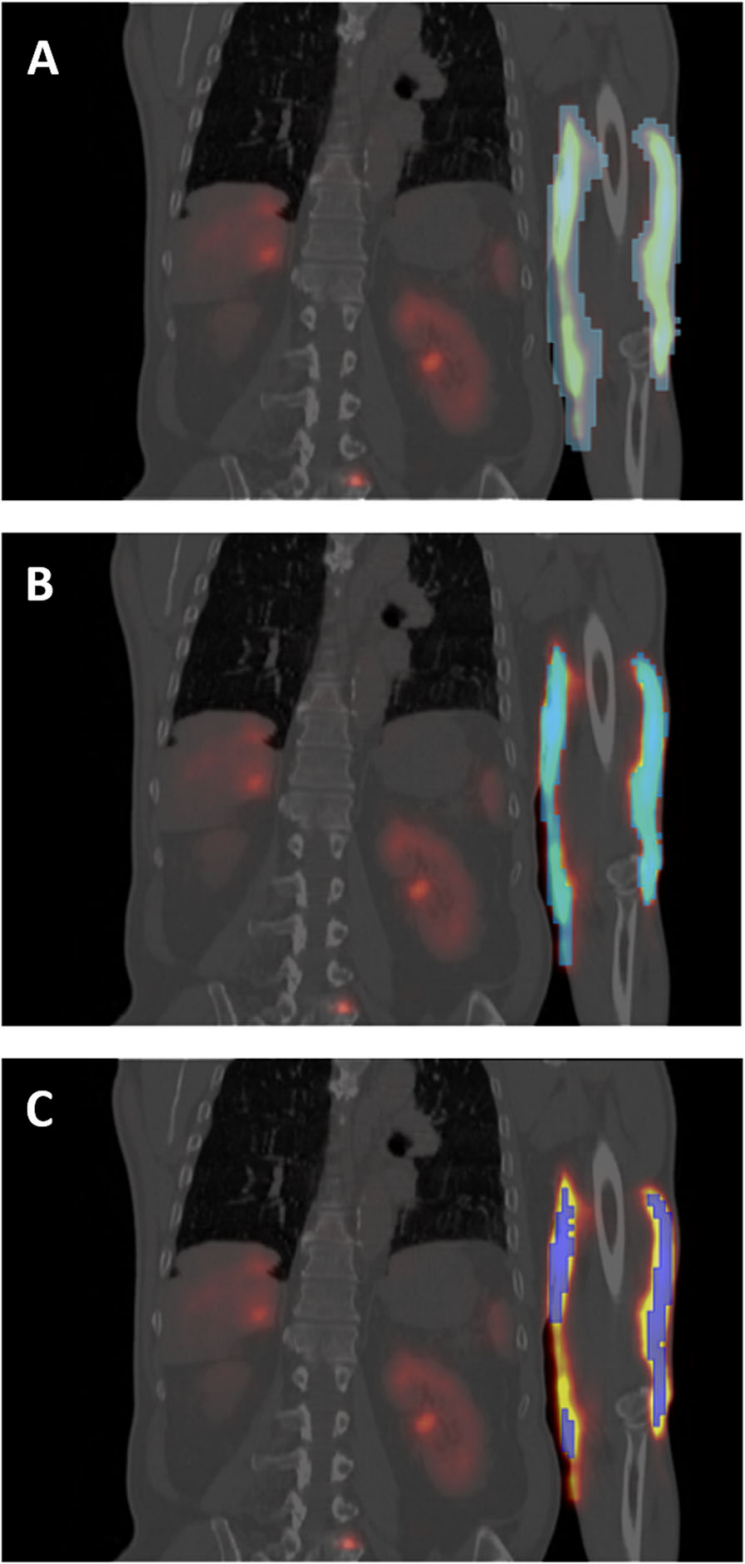
Coronal slice of *T*_5h_ SPECT/CT with superimposed VOI corresponding to 1000 mL (**a**), 400 mL (**b**), and 180 mL (**c**) VOIs

The volumes of the 3 threshold-based VOI *V*_1_, *V*_2_, and *V*_3_ were respectively 1000 mL, 400 mL and 180 mL, their uncertainty was not accounted for in the total uncertainty determination. These VOI are shown on the *T*_5h_ SPECT/CT (Fig. 8). These VOI were drawn on the *T*_5h_ SPECT/CT and copied on the *T*_20h_ and *T*_26h  _ SPECT images. Their positions were slightly adjusted to obtain the maximum number of counts in each VOI.

**Fig. 9:**
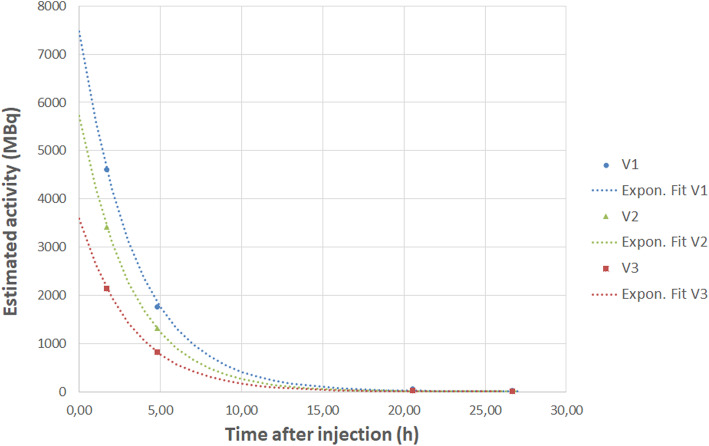
Estimated time activity curves for the 3 VOI of 1000, 400, and 180 mL

Time activity curves in the extravasation area for the 3 VOI are plotted in Fig. 9. Monoexponential curves following Eq. (2) model were fitted to the data to estimate effective half-life with parameters shown in Table 4, where *A*(*t*) is the activity in the arm, *A*_0_ is the estimated activity at *T*_0_ and *T*_eff_ the effective half-life. Due to the higher uncertainty of the activity determined at *T*_2h_, a non-linear regression with unequal weighting factor was used to determine *A*_0_ and *T*_eff_ and their uncertainty using Matlab® Statistic toolbox.

2 $$ A(t)={A}_0{e}^{-\ln (2)t/{T}_{eff}} $$

**Table 4:** Estimation of effective half-life, activity at infusion time, and corresponding local absorbed dose for volumes *V*_1_, *V*_2_, and *V*_3_

	Effective half-life *T*_eff_ (h)	*A*_0_ (MBq)	Estimated dose (Gy)
*V*_1_	2.39 ± 0.002	7471 ± 26	2.26 ± 0.07
*V*_2_	2.257 ± 0.002	5715 ± 20	4.07 ± 0.13
*V*_3_	2.256 ± 0.002	3592 ± 13	6.77 ± 0.22

*A*_0_ is a purely theoretical value, as *T*_0_ corresponds to the beginning of the infusion. The infusion process is slow and the activity cannot be instantaneously spread in the extravasated area. It nevertheless gives an idea of the amount of activity considered in each of the 3 dose estimations.

Based on these models, and using the mean dose coefficient of 24.35 nGy kg/(MBq s), estimated local dose ranged from 2.26 to 6.77 Gy, according to the volume considered (Table 4).

## Discussion

### Effects of extravasation

The range of dose leading to deterministic effects depends on the type of radiation and the exposed organ. For skin, it is well reported for external exposure, such as fluoroscopy [16]. For extravasation cases, a few studies linked dose values to observed effects. Shapiro et al. reported a threshold dose of 20 Gy to observe radiation induced injury in for extravasation of radiopharmaceutical in interstitial tissue [15]. Bonta et al. reported skin injury and desquamation with a dose between 22 and 36 Gy during an extravasation of 131I-Metaiodobenzylguanidine [17]. Williams et al. reported wet desquamation with a dose between 20 and 40 Gy during an extravasation of Yttrium-90-Ibritumomab Tiuxetan [18].

Our estimation of absorbed dose in the subcutaneous tissue in the arm of the patient is comprised between 2 and 7 Gy. This dose is in the same range than another extravasation case published by Arveshoug et al., who report an absorbed dose of 6 Gy in the arm in a similar case. We can hypothesize that local absorbed skin dose (especially hypodermic dose) is similar to subcutaneous tissue dose (Fig. 8). ICRP reported a threshold between 3 and 6 Gy for deterministic effects for the skin; this value corresponds to the dose level where 1% of exposed persons would experience the effect [19].

The patient was seen the day after, 6 days, and 3 weeks after the incident. He did not show any clinical sign of irradiation, like erythema, and did not report any pain in the area of extravasation. Although the uncertainties in our calculated doses are high, the results were sufficiently accurate enough to estimate the risk.

### Comparison of dose estimations

We performed two dose estimations: a first estimation in the evening and another more accurate estimation in the days following the incident. The first dose evaluation was necessary to rapidly evaluate the need of a surgical intervention. The purpose of the second dose estimation was to get a more accurate result to confront with the patient follow-up. Dosimetric data for extravasation cases are scarce in the literature [4]. At the time of the incident, no data has been published for ^177^Lu-DOTATATE, making it difficult to estimate the consequences of the extravasation without dosimetric evaluation.

The second activity estimation relied on the calibration factor determined on the SPECT phantom study. A different approach to estimate the activity or even verify our calculations could have been to make the assumption that the total activity of the patient was in the first whole body image counts and use a ratio between the counts in the arm and in the whole body with an estimation of the volume of interest made on SPECT/CT images. Unfortunately, the patient urinated before its first whole body acquisition. With no knowledge of the remaining activity in the whole body, we could not base our estimation on this approach. Retrospectively, the percentages of activity in the arm calculated using the whole body images (74% at *T*_2h_) and SPECT images (69% at *T*_0_) are very close. If we hypothesized that the first miction activity was negligible and that the first whole body image represents the total activity administered to the patient, the error on the dose estimation would have been very low, compared to the overall dose uncertainty.

### Distribution of ^177^Lu-DOTATATE after extravasation

Whole body images show the efficiency of adapted interventions and lymphatic drainage, reducing extravasation in patient, with a rather short effective half-life of around 3 h.

Specific interventions were applied to the patient, which were detailed in our local procedure for extravasation cases. After the incident, we completed this document with the acquisitions of WB and SPECT/CT images as quickly as possible after the detection of the incident. Images should be acquired at least twice a day, as it has been done for this patient.

The distribution of ^177^Lu-DOTATATE in the patient the day after the injection (Fig. 7) shows a classical pattern of distribution for GEP NET patients treated with ^177^Lu-DOTATATE. After lymphatic drainage, the product distributed in blood flow and accumulated in tumors and metastases of the patient. The impact of extravasation on the efficiency of the treatment is hard to evaluate but most of the product in all likelihood reached its target. Furthermore, after having completed the treatment (with administration of 3 subsequent cycles of Lutathera), the patient reported a significant improvement of carcinoid syndrome with disappearance of flush, and diminution of diarrhea. Tumoral disease remained stable 18 months after the end of PRRT.

### Limits of the dose estimation

The dose estimation resulting from the extravasation has several limits. The determination of the volume used for dose calculation was challenging, due to its complex shape and its decrease over time. We therefore chose 3 volumes from 480 mL to 1 L, to get a plausible range of absorbed dose and an idea of the dose distribution in the extravasated area. The dose factor used assumed a spherical shape, which was not the case on the images. However, we choose to use these factors, given their small variation according to the volume of the sphere as mentioned earlier. A voxel-based approach for dose determination with a more refined dose calculation model, such as a convolution-based method for instance, could have been useful in this case to characterize the limits of this approach. A dose-volume histogram would have been also very useful to determine more accurately the dose distribution. Unfortunately, a voxel-based dosimetry software is not yet routinely available in the center.

Several corrections have not been applied to the images: the deadtime effect has not been taken into account, and counts from the arm VOI were not corrected for partial volume effect. Data were not available in the center to estimate the impact of these two effects on the quantification of ^177^Lu SPECT images. Moreover, the complex shape of the extravasated volume made a robust partial volume effect correction very difficult. The uncertainty of the dose estimation is also underestimated: the uncertainty of the count rate was evaluate assuming Poisson statistics, without taking into account the VOI delineation and recovery coefficient uncertainty as mentioned in [10], as data required by this approach were not available for our system.

A recent study by Uribe et al. evaluated the deadtime for ^177^Lu on a Symbia system [20] and reported a 10% count loss for images acquired 3 h after injection for patients treated with 7.4 GBq of ^177^Lu, and no count loss for images acquired later. These data have been measured on a 3/8″ crystal camera and cannot be used to correct for deadtime on our data. Nevertheless, taking deadtime into account would have led to a slightly increased activity estimation on *T*_2h_ images, and a slightly decreased effective half-life. The resulting impact on dose estimation would be a few percent decreases.

The shape of the time activity curve could also be refined. Given its short half-life, the accuracy of the fit would have been better with some earlier time points. For instance, an uptake phase could have been modeled to consider the infusion process. This is very difficult, as the delivery of the radiopharmaceutical is not linear with time. A saline solution is injected in the vial and this combination is pushed in the vein of the patient. The flow rate of the saline solution is not equal to the flow rate of the radiopharmaceutical. With this method, the majority of the product is delivered during the first minutes of the infusion process and the rest of the infusion consists in a rinsing phase.

Despite these limitations, the dose estimation is consistent with the absence of observable effects.

## Conclusion

Local dose was estimated for an extravasation case in the arm of a patient during a ^177^Lu-DOTATATE infusion, using SPECT/CT, WB images, and a quantitative procedure, initially developed for renal dosimetry. The estimated dose to the tissue in the patient’s arm, comprised between 2 and 7 Gy based on the VOI considered to define the extravasated volume, is in line with the absence of detectable effects. Adapted interventions promoted the elimination of ^177^Lu-DOTATATE in the arm and limited the absorbed dose in non-target tissues.

## Data Availability

The datasets generated and analyzed during the current study are available from the corresponding author on reasonable request.
